# Temporal trends in hospitalisations for venous thromboembolic events in England: a population-level analysis

**DOI:** 10.1136/bmjopen-2024-090301

**Published:** 2025-03-29

**Authors:** Mark Hughes, Mark D Russell, Ritika Roy, Daksh Mehta, Sam Norton, Fabiola Atzeni, James B Galloway

**Affiliations:** 1 Centre for Rheumatic Diseases, King’s College London, London, UK; 2 Psychology Department, Insitute of Psychiatry, King’s College London, London, UK; 3 Rheumatology Unit, University of Messina, Messina, Italy

**Keywords:** Thromboembolism, COVID-19, EPIDEMIOLOGY, Primary Health Care, PUBLIC HEALTH, RHEUMATOLOGY

## Abstract

**Abstract:**

**Objectives:**

To describe temporal trends in hospitalisation episodes for venous thromboembolic events (VTEs) in England, and compare hospitalisation rates for pulmonary emboli (PEs) and deep vein thrombosis (DVT).

**Methods:**

Retrospective observational study.

**Setting:**

Secondary care in England, UK, between April 1998 and March 2022.

**Participants:**

Individuals with hospitalisations for VTE recorded in the NHS Digital Hospital Episode Statistics dataset.

**Primary and secondary outcomes:**

The primary outcome was temporal trends in hospitalisation episodes for PE, DVT and VTE overall between 1 April 1998 and 31 March 2022. Secondary outcomes included the proportion of all-cause hospital admissions that were due to VTE; the proportion of all VTE hospitalisations that were recorded as primary admission diagnoses; the male/female split in hospitalisation episodes for VTE; and temporal changes in hospitalisation rates by age.

**Results:**

Between 1998 and 2022, hospitalisations for VTE increased by 62.6%, from 109.5 to 178.1 per 100 000 population. This was driven by a 202% increase in hospitalisations for PE (from 40.4 to 122.2 per 100 000 population). In contrast, hospitalisations for DVT decreased by 19.1% over this period (from 69.1 to 55.9 per 100 000 population). Overall, VTE remained stable as a proportion of all-cause hospital admissions between 1998/1999 and 2019/2020 (0.45% and 0.43%, respectively), before increasing after the onset of the COVID-19 pandemic in England (0.59% in 2020/2021 and 0.51% in 2021/2022).

**Conclusion:**

Hospitalisations for VTE increased markedly in England between 1998 and 2022, driven by large increases in hospitalisations for PE. In contrast, hospitalisations for DVT decreased overall, which may reflect the success of primary care DVT management pathways. Our findings suggest that preventative measures are needed to reduce the incidence of hospitalisations for PE.

**STRENGTHS AND LIMITATIONS OF THIS STUDY**

- Population-level data for all hospitalisation events in England provided us with reliable estimates of admissions for venous thromboembolic events.
- Data were available since 1998, facilitating detailed analyses of temporal trends over a more than 20-year period.
- We were able to explore trends in primary vs secondary hospitalisation events, and investigate age- and sex-related changes over the study period.
- As with other analyses of aggregated health data, we are unable to definitively say whether hospitalisation trends were due to underlying incidence changes, service-related changes or changes in coding practices.

## Introduction

 Venous thromboembolism event (VTE) is a life-threatening condition, characterised by the presence of thrombi within veins. VTE includes deep vein thrombosis (DVT) and pulmonary embolism (PE). VTE is complex and multifactorial in nature, with risk factors including older age, malignancy, fracture, immobility, obesity, smoking and a personal or familial history of VTE.^1^

VTE is one of the leading cardiovascular causes of death globally, behind coronary heart disease and ischaemic stroke.^2^ Despite treatments such as warfarin, heparin and direct oral anticoagulants (DOACs),^3^ the mortality rate from VTE has been estimated at 21.7 per 100 000 in the UK.^4^ An improved understanding of the epidemiology of VTE is essential if public health interventions are to be implemented to reduce morbidity and mortality from this condition.

There are numerous factors that can influence the burden of hospitalisations for VTE. Primary care-led pathways to manage DVTs in the community have been introduced throughout the UK,^5^ with the aim of reducing the need for hospitalisation; however, their success in doing so has not been evaluated previously at a population level. In contrast, risk factors for VTE, such as obesity, have become more prevalent in recent years.^6^ Additionally, VTE is a well-recognised complication of COVID-19 infection.^7^ Data are lacking on how the overall burden of hospitalisations for VTE has changed in light of these factors.

Our objective was to use population-level data in England to describe temporal trends in hospitalisations for VTE between 1998 and 2022. We explored the relative contributions of DVTs and PEs to the overall burden of hospitalisations for VTE, and described the impact of the COVID-19 pandemic on VTE hospitalisations.

## Materials and methods

### Study type and data sources

We conducted a population-level observational study to describe hospitalisations for VTE in England between 1 April 1998 and 31 March 2022. We used two publicly available, population-level datasets in England: the Office for National Statistics dataset,^8^ containing population estimates for England (published in June of each year), and the NHS Digital Hospital Episode Statistics (HES) dataset.^9^ Within NHS Digital HES, the Admitted Patient Care (APC) dataset includes data on all admissions to NHS hospitals in England for a given year. Aggregated data are reported annually (covering a period from 1 April to 31 March), containing coded information on primary and secondary admission diagnoses. Admissions are reported as finished consultant episodes, which refer to a single episode of care provided by a consultant during admission, and finished admission episodes, which refer to a single admission episode from admission to discharge (ie, potentially including multiple finished consultant episodes). Coded data on primary admission diagnoses of VTE (ie, where VTE was the primary diagnosis for that admission) were available in HES APC from 1 April 1998 to 31 March 2022. Additionally, aggregated data on the number of finished consultant episodes with secondary admission diagnoses of VTE (ie, where VTE was a contributory diagnosis for that admission) were available in HES APC from 1 April 2012 to 31 March 2022.

### Diagnostic coding inclusion and exclusions

The diagnostic codes for VTE in this study were coded according to the 10th version of the International Classification of Diseases (table 1). An a priori decision was made not to include other forms of VTE, such as cerebral venous sinus thrombosis, due to the rarity of these events, relative to DVT and PE.

**Table 1:** ICD descriptions of the included venous thromboembolic event conditions and their respective ICD codes

ICD description	ICD code
Phlebitis and thrombophlebitis of femoral vein	I80.1
Phlebitis and thrombophlebitis of other deep vessels of lower extremities	I80.2
Phlebitis and thrombophlebitis of lower extremities, unspecified	I80.3
Deep phlebothrombosis in pregnancy	O22.4
Deep phlebothrombosis in the puerperium	O87.1
Pulmonary embolism with mention of acute cor pulmonale	I26.0
Pulmonary embolism without mention of acute cor pulmonale	I26.9

ICDInternational Classification of Diseases

### Statistical analysis

For each year of the study period, we estimated rates (per 100 000 population) of hospitalisations with primary admission diagnoses of DVT or PE. This was calculated by dividing the number of finished consultant episodes with primary admission diagnoses of DVT or PE in England by the mid-year population estimate for England for that year. Additionally, we estimated yearly hospitalisation rates for VTE overall, which combined hospitalisations for DVT and PE. We presented temporal trends in hospitalisation rates in tabular form, and graphically using scatter plots. Joinpoint regression was implemented using a segmented regression approach with a grid search across breakpoints allowing for one and two joinpoints, to understand temporal causes of the changes observed. The best-fitting model was identified using the lowest Bayesian information criterion and included comparison to a linear model with no breakpoints. As sensitivity analyses, we reported hospitalisation rates for DVT, PE and VTE using finished admission episodes, instead of finished consultant episodes, to account for admissions involving multiple consultant episodes with the potential for repeat counting of VTE events.

To account for increases in the number of all-cause hospitalisations over the study period, we reported the proportion of all-cause hospitalisation episodes that had primary admission diagnoses of DVT, PE and VTE overall. As secondary analyses, we presented temporal trends in the proportion of all admission diagnoses for DVT, PE and VTE (ie, combined primary and secondary admission diagnoses) that were primary admission diagnoses. These data were available in HES APC from 1 April 2012 onwards. Additionally, we reported the male/female split in the proportion of primary admission diagnoses due to DVT, PE and VTE overall for each year of the study period, as well as the hospitalisation rates for males and females separately.

To investigate temporal changes in hospitalisation rates by age, we reported: (a) the mean age at hospitalisation for VTE, DVT and PE over the study period; (b) age-stratified rates for the age groups 0–14, 15–59, 60–74 and 75+ years (reflective of the data available in the HES datasets); and (c) age-standardised rates for the years 2000, 2010 and 2020, which we calculated by dividing the number of finished consultant episodes with primary admission diagnoses of DVT or PE in England by the mid-year population estimate for England for that year, for each age group. The mean age was provided within the HES datasets for each year. Age was directly standardised for, using a weighted average of the stratum-specific rates relative to the year 2000. This enabled direct comparison with reduced confounding from age. All data management and statistical analyses were conducted using Stata V.17 (StataCorp).

## Results

### Hospitalisations with primary admission diagnoses of VTE

**Figure 1:**
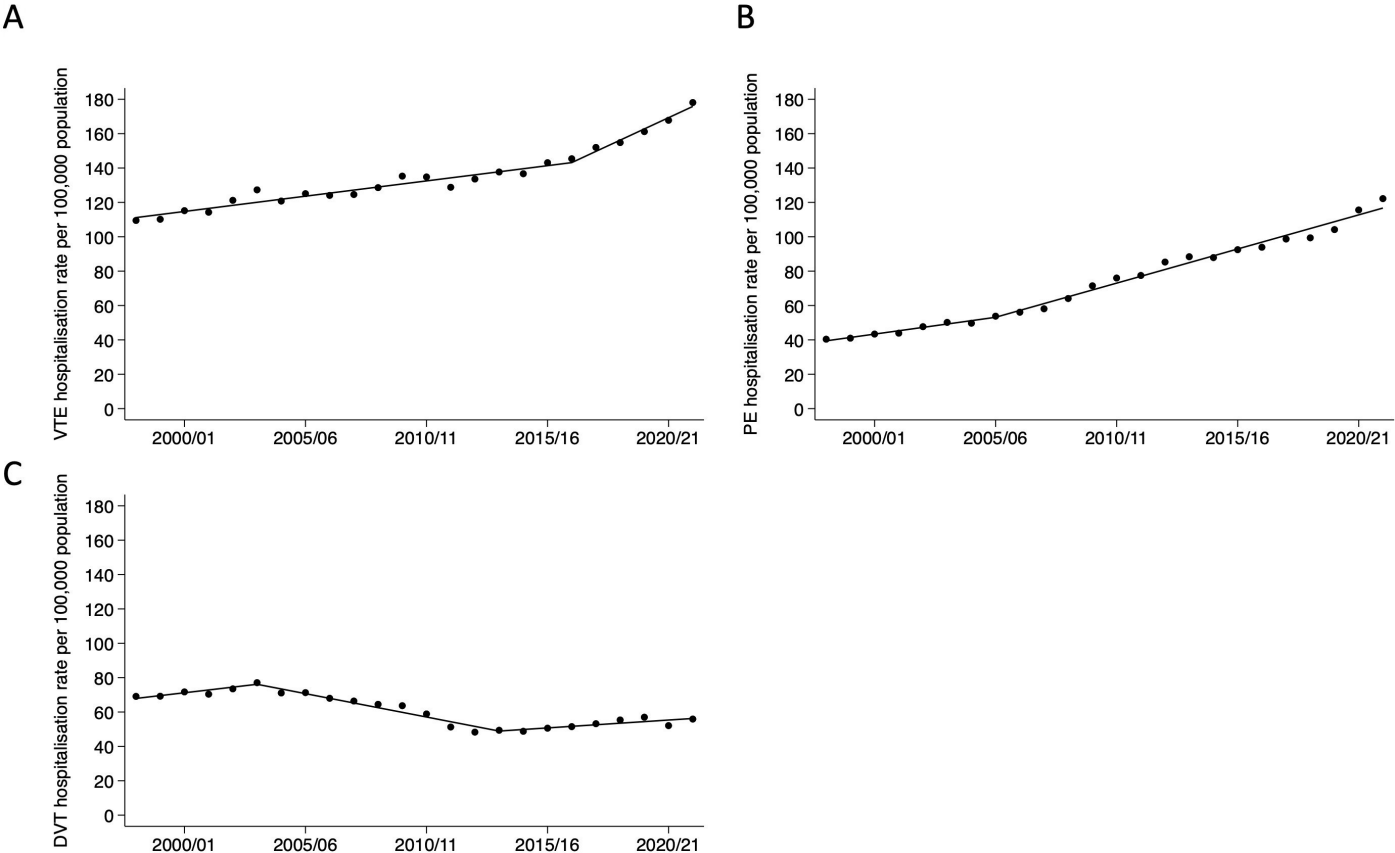
Joinpoint regression using a segmented regression with an automated search across breakpoints exploring temporal changes for (**A**) VTE (DVT and PE combined), (**B**) PE and (**C**) DVT in England between 1998 and 2022. DVT, deep vein thrombosis; PE, pulmonary embolism; VTE, venous thromboembolic event.

Between 1 April 1998 and 31 March 2022, there was a 62.6% increase in the rate of hospitalisations with primary admission diagnoses of VTE, from 109.5 to 178.1 admissions per 100 000 population, respectively (figure 1A and table 2). For PEs, hospitalisations increased by 202%, from 40.4 to 122.2 per 100 000 population (figure 1B). Hospitalisations for DVTs decreased by 19.1%, from 69.1 to 55.9 per 100 000 population (figure 1C); a non-linear temporal relationship was observed: between 2003/2004 and 2012/2013, hospitalisations for DVTs decreased from 77.1 to 48.3 per 100 000 population; from 2012/2013 onwards, there was an increase in DVT hospitalisations (from 48.3 to 55.9 per 100,000) (figure 1C). Sensitivity analyses were performed to evaluate changes in finished admission episodes (as opposed to finished consultant episodes) due to VTE, with similar trends observed (online supplemental figure 1 and table 1).

**Table 2:** Number of admissions (finished consultant episodes) for VTE (DVT and PE combined), PE and DVT, and corresponding rates per 100 000 population in England between 1998 and 2022

Year	VTE admission episodes (n)	VTE hospitalisation rate (per 100 000 population)	PE admission episodes (n)	PE hospitalisation rate (per 100 000 population)	DVT admission episodes (n)	DVT hospitalisation rate (per 100 000 population)
1998/1999	53 473	109.5	19 739	40.4	33 734	69.1
1999/2000	54 038	110.2	20 093	41.0	33 945	69.2
2000/2001	56 703	115.2	21 379	43.4	35 324	71.7
2001/2002	56 533	114.3	21 705	43.9	34 828	70.4
2002/2003	60 197	121.2	23 699	47.7	36 498	73.5
2003/2004	63 569	127.3	25 062	50.2	38 507	77.1
2004/2005	60 655	120.8	24 951	49.7	35 704	71.1
2005/2006	63 304	125.1	27 205	53.8	36 099	71.3
2006/2007	63 258	124.1	28 611	56.1	34 647	68.0
2007/2008	64 008	124.6	29 877	58.1	34 131	66.4
2008/2009	66 656	128.6	33 231	64.1	33 425	64.5
2009/2010	70 603	135.3	37 333	71.5	33 270	63.7
2010/2011	70 974	134.8	39 987	76.0	30 987	58.9
2011/2012	68 414	128.8	41 176	77.5	27 238	51.3
2012/2013	71 490	133.6	45 626	85.3	25 864	48.3
2013/2014	74 183	137.7	47 594	88.4	26 589	49.4
2014/2015	74 264	136.7	47 734	87.9	26 530	48.8
2015/2016	78 426	143.1	50 696	92.5	27 730	50.6
2016/2017	80 373	145.4	51 894	93.9	28 479	51.5
2017/2018	84 532	152.0	54 919	98.7	29 613	53.2
2018/2019	86 647	154.8	55 626	99.4	31 021	55.4
2019/2020	90 712	161.2	58 636	104.2	32 076	57.0
2020/2021	94 874	167.8	65 389	115.6	29 485	52.1
2021/2022	100 665	178.1	69 064	122.2	31 601	55.9

DVTdeep vein thrombosisPEpulmonary embolismVTEvenous thromboembolic event

### VTE as a proportion of all-cause admissions

**Figure 2:**
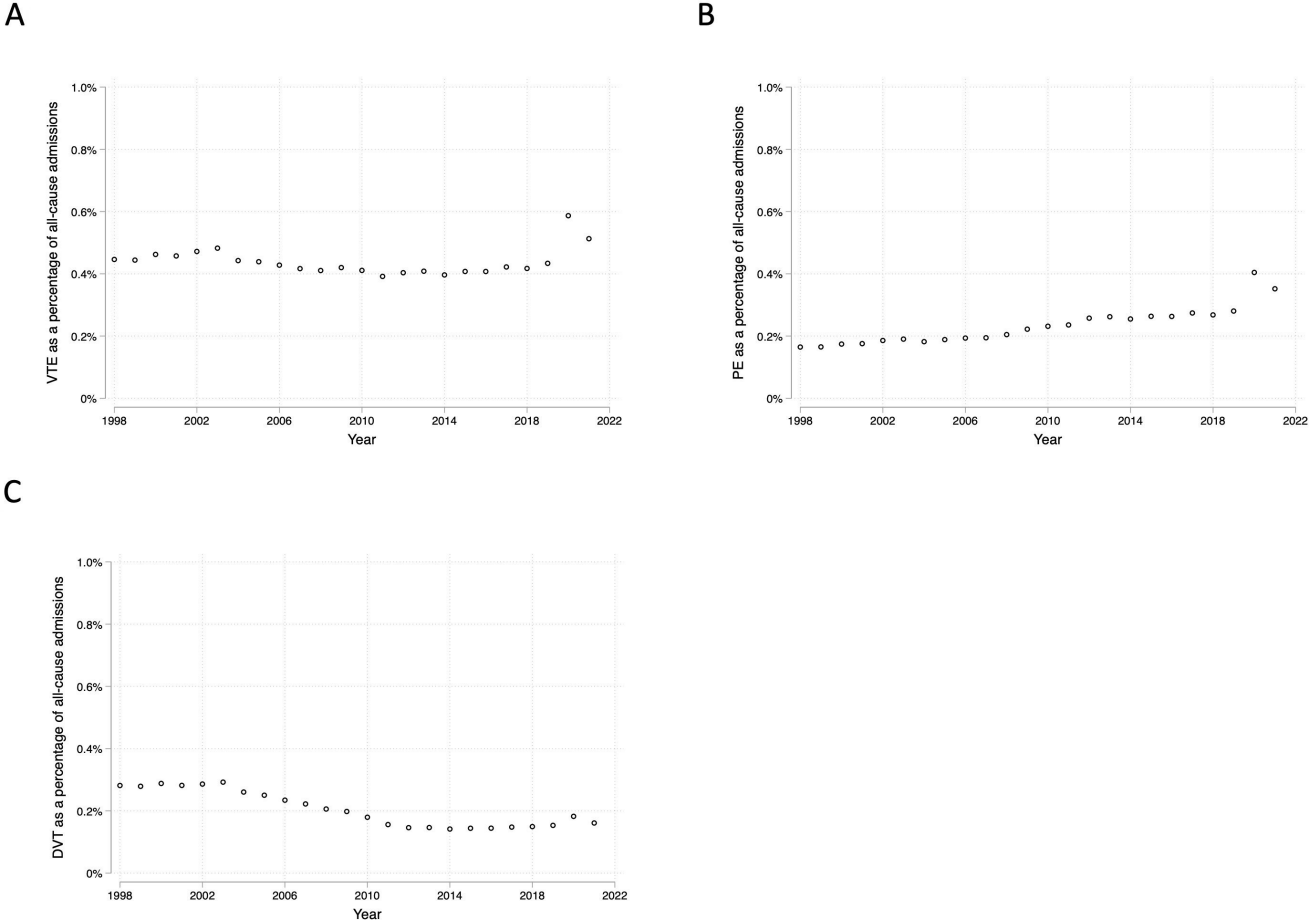
Primary admission diagnoses of (**A**) VTE, (**B**) PE and (**C**) DVT as a percentage of all-cause hospital admissions between 1998 and 2022. DVT, deep vein thrombosis; PE, pulmonary embolism; VTE, venous thromboembolic event.

To account for changes in the number of all-cause admissions over the study period, we explored what proportion of all-cause admissions were due to VTE. The total number of all-cause admissions in England increased by 74.5% between 1998/1999 and 2019/2020, from 11 983 893 admissions to 20 912 276 admissions (online supplemental table 2). This was followed by a decrease in the number of all-cause admissions in 2020/2021 (corresponding to the onset of the COVID-19 pandemic), to 16 168 689 admissions, followed by a partial recovery in 2021/2022, to 19 626 344 admissions. As a proportion of all-cause admissions, VTE remained relatively stable between 1998/1999 and 2019/2020 (0.45% and 0.43%, respectively) (figure 2A). In 2020/2021, the proportion of all-cause admissions due to VTE increased to 0.59%, before decreasing in 2021/2022 to 0.51%. PEs increased as a proportion of all-cause admissions from 1998/1999 to 2019/2020 (from 0.16% to 0.28%, respectively), followed by a further marked increase after the onset of the COVID-19 pandemic: 2020/2021 (0.40%); 2021/2022 (0.35%) (figure 2B). DVTs decreased as a proportion of all-cause admissions between 1998/1999 and 2019/2020 (from 0.28% to 0.15%, respectively). In 2020/2021, there was a marginal increase in DVTs, to 0.18%, followed by a relative reduction, to 0.16%, in 2021/2022 (figure 2C).

### Hospitalisations with primary or secondary admission diagnoses of VTE

Data on secondary admission diagnoses were available from 2012 onwards. Since 2012, primary VTE admissions decreased as a proportion of all VTE admissions (ie, primary and secondary admission diagnoses combined), from 53.0% in 2012/2013 to 44.4% in 2021/2022 (online supplemental table 3). A similar pattern was observed for PE and DVT separately: primary PE admissions decreased as a proportion of all PE admissions, from 57.7% in 2012/2013 to 45.7% in 2021/2022 (online supplemental table 4); primary DVT admissions decreased as a proportion of all DVT admissions, from 46.4% in 2012/2013 to 41.7% in 2021/2022 (online supplemental table 5).

### Differences in admissions due to VTE by gender and age

**Figure 3:**
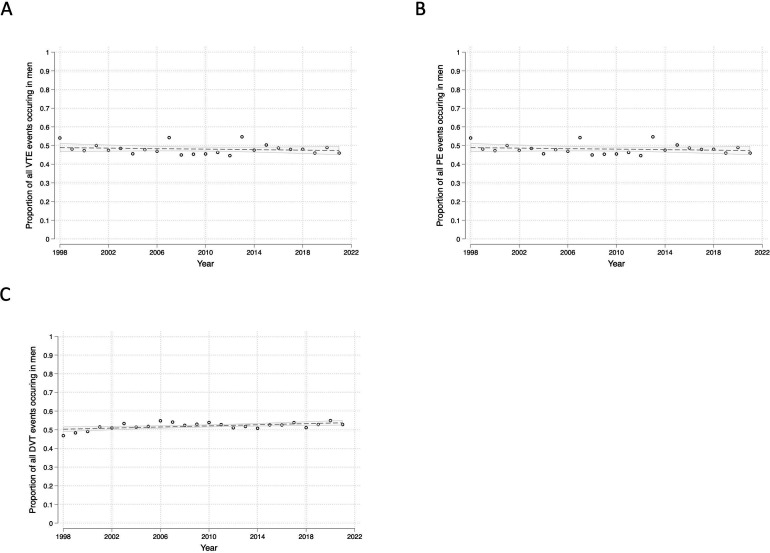
Proportion of (**A**) VTE, (**B**) PE and (**C**) DVT admissions that occurred in males versus females between 1998 and 2022. DVT, deep vein thrombosis; PE, pulmonary embolism; VTE, venous thromboembolic event.

Between 1998 and 2022, the proportion of hospitalisations with primary admission diagnoses of VTE occurring in males and females remained close to 1:1 (figure 3A and online supplemental figure 2A). The same was true of DVTs and PEs individually (figure 3B,C and online supplemental figure 2B,C).

**Figure 4:**
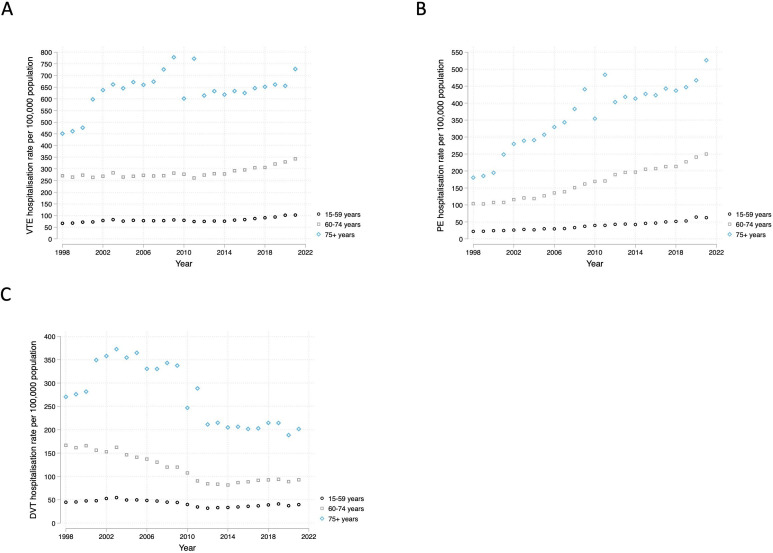
Hospitalisation rate of (**A**) VTE, (**B**) PE and (**C**) DVT admissions separated by age groups 15–59, 60–74 and 75+ between 1998 and 2022. DVT, deep vein thrombosis; PE, pulmonary embolism; VTE, venous thromboembolic event.

The mean age at hospitalisation for people with VTE, DVT or PE remained stable over the study period (online supplemental figure 3). Hospitalisation rates for VTE overall, and DVT and PE separately, were highest in the 75 years and above age group, followed by the 60–74 age group, then the 15–59 age group (figure 4). Increases in PE and VTE hospitalisations overall were observed in all three age groups, contrasting decreases in DVT hospitalisations. Age-standardised rates for VTE, PE and DVT are shown in online supplemental figure 4.

## Discussion

Between 1998 and 2022, the hospitalisation rates for VTE increased by 63% in England. This was driven by a tripling of hospitalisations for PE over the study period. In contrast, hospitalisations for DVTs decreased by 19% between 1998 and 2022, with much of this decrease occurring prior to 2012. These patterns remained consistent when we accounted for increases in all-cause admissions over time.

To our knowledge, this is the first population-level study to report on temporal trends in hospitalisations for VTE in the UK, and to describe the relative contribution of DVT and PE. Studies from other countries have reported more broadly on the incidence of VTE (including hospitalised and non-hospitalised events), with comparable findings.^10^,^13^ A study from Tromsø in Norway, from 1996/1997 to 2010/2011, reported that the incidence rate of PE increased from 45 per 100 000 person-years to 113 per 100 000 person-years, respectively, whereas DVT incidence rates decreased from 112 per 100 000 person-years to 88 per 100 000 person-years.^10^ A study from Worcester, USA, showed that age- and sex-adjusted, first-time VTE event rates increased from 73 per 100 000 in 1985/1986 to 133 per 100 000 in 2009, which was mostly attributable to increasing PE hospitalisation rates.^11^ A study in Denmark between 2006 and 2015 showed an increase in PE incidence from 4.6 per 10 000 to 9.0 per 10 000, whereas for DVT the rate decreased from 7.9 per 10 000 to 7.6 per 10 000.^12^ Similarly, a study in Brazilian older adults reported an increase in PE hospitalisations between 2010 and 2019, contrasting a decrease in DVT hospitalisations.^13^

One possible explanation for our findings could be that the underlying incidence of VTE has changed over time, resulting in the observed patterns of hospitalisations. Some risk factors for VTE, such as obesity,^6 14^ have increased in prevalence in recent years^15^; however, this would not explain the disparity between increasing PE hospitalisations and decreasing DVT hospitalisations. Among cardiovascular risk factors, hypertension has shown a protective effect for future VTE risk,^16^ but a positive association between hypertension and PE.^17^ Hypertension prevalence remained broadly stable between 2003 and 2014 and decreased modestly until 2019, suggesting this was unlikely to have influenced our observed trends.^18^ The trends we observed for hospitalisation rates for VTE contrast with conditions such as myocardial infarction, which has shown decreasing hospitalisation rates over the last few decades. One study reported a reduction of 31% and 14% in men and women, respectively, from 1968 to 2016.^19^ We found that the mean age at hospitalisation remained similar for VTE, DVT and PE over the study period, which would go against population ageing being the primary driver of the increase in VTE hospitalisations over the study period, supported by comparable findings from our age-standardised analyses.

We observed an increase in hospitalisations for PE after the start of the COVID-19 pandemic. PE is a recognised complication of COVID-19,^20^ which may have contributed to the increase in PE admissions after 2020. Another potential explanation for the observed increase in PE over the study period could be improved access to imaging modalities, such as CT pulmonary angiograms (CTPAs). Data from the UK HES show that the number of CTPAs performed during all NHS hospitalisation episodes increased from 86 397 to 166 341 between 2012/2013 and 2021/2022.^9^

The contrasting trends in hospitalisations for PE and DVT may also relate to changes in where these conditions are diagnosed and managed. While the majority of PEs are diagnosed and managed in hospital, there has been a concerted effort in the UK to manage DVTs in primary care. Community-based pathways have been introduced throughout the UK over the last two decades, to support primary care-based investigation (eg, D-dimer blood tests and Doppler ultrasound) and management (eg, using DOACs).^21^ Our finding of decreasing DVT hospitalisations might therefore represent the success of these programmes in managing DVTs in the community. Of note, however, the decrease in DVT hospitalisations during our study period was not linear: a decreasing trend in DVT hospitalisations was observed prior to 2012; after 2012, DVT hospitalisations increased modestly. Further research is needed to further understand this pattern. One possible interpretation is that community-based pathways may have been effective at reducing DVT hospitalisations early in our study period, followed by subsequent increases in underlying DVT incidence (eg, due to risk factors such as obesity). Additionally, DOAC use has increased significantly, from 0% in 2000–2002 to 47.3% in 2017–2019, whereas warfarin experienced a sharp decrease in use over the same time period (from 58.1% to 6.6%, respectively), resulting in a slight decline in the overall proportion of individuals with VTE who were initiated on anticoagulants.^22^ A reduction in the proportion of individuals with VTE who received anticoagulant therapy could potentially have contributed to the temporal trends observed in our study, for example, through recurrent VTE events and/or progression of DVT to PE. While we were unable to explore trends in the proportion of hospitalisation events in our study that were from incident vs recurrent VTE events due to the aggregated data sources used, this certainly warrants further investigation in future studies.

To further investigate the relative contribution of incidence changes versus service-related factors on VTE hospitalisations, we evaluated changes in hospitalisations with secondary admission diagnoses of DVT or PE. Whereas admissions with primary admission diagnoses of DVT would be expected to decrease if community-based pathways were implemented effectively, admissions where DVTs occurred as secondary diagnoses (eg, during admissions for surgery) would be less influenced by primary care pathways. Although data on secondary admissions were only available from 2012 onwards, we showed a similar pattern for both DVT and PE, with primary admission diagnoses decreasing as a proportion of combined primary/secondary admission diagnoses. Additionally, we found that the male/female split in VTE hospitalisations remained similar over the study period, as was the mean age at hospitalisation. Despite the number of people aged 75 and over increasing by one-third in England between 2000 and 2020, we observed comparable trends between our primary analysis and our age-standardised analyses. Together, this suggests that service and/or management-related changes are likely to have contributed more to the observed trends in hospitalisations than underlying pathophysiological changes.

Our findings have strong implications from a public health perspective. VTE has substantial health and economic costs, contributing to longer hospital stays, short- and long-term complications, and mortality. A study in the USA reported that death occurred in 6% and 12% of DVT and PE cases, respectively, within 1 month of diagnosis.^23^ There are highly effective preventative treatments for VTE, in the form of thromboprophylaxis,^24^ and there have been extensive efforts to implement VTE risk assessments in at-risk patients (eg, during hospitalisations and after surgery).^25^ Our findings of increasing numbers of hospitalisations for PE (and VTE overall) suggest that these preventative measures need to be implemented more widely. A UK primary-care based study reported that 95% of GPs and practice nurses never or only occasionally performed VTE risk assessments, and that 79% never or only occasionally provided advice about VTE risk to patients prior to elective hospital admissions.^26^

The strengths of our study include the population-level coverage of the datasets used. The NHS Digital HES APC dataset captures data on all admissions to NHS hospitals in England, providing us with reliable estimates of VTE hospitalisations. Additionally, we evaluated changes in hospitalisations in England over a more than 20-year period, enabling us to describe temporal trends in detail.

There are also limitations to our study. As with other epidemiological studies using aggregated coded health data, case verification was not possible, leading to a potential for diagnostic misclassification. We were unable to determine whether changing hospitalisation patterns were due to underlying incidence changes, service-related changes or changes in coding practices. Similarly, we were unable to separate out incident VTE admission events from recurrent VTE admissions, which precluded further investigation of whether there were missed opportunities for primary VTE prevention (eg, due to risk factor management) and/or missed opportunities for secondary prevention (eg, due to inadequate treatment). As we only captured hospital admission data, our findings cannot be generalised to changes in VTE incidence overall; for example, cases diagnosed and managed solely in primary care would not be captured. We only had aggregated data available for analysis; future analyses using individual-level data will help to explore other important predictors of VTE risk, such as comorbidities and the influence of medications (such as anticoagulant prescribing practices). We were unable to present age-stratified rates by more granular age bands due to a lack of these data in earlier datasets. Finally, as the dataset encompassed admissions in England only, our findings should not be assumed to be generalisable to other countries.

In conclusion, we showed that hospitalisations for VTE have increased markedly over the last 20 years. This has been driven by increases in hospitalisations for PE, contrasting an overall decrease in hospitalisations for DVT. While the decrease in DVT hospitalisations may relate to successful implementation of primary care-based management pathways, our data suggest that there is a need for more widespread implementation of preventative measures to reduce hospitalisations for PE.

## supplementary material

10.1136/bmjopen-2024-090301 online supplemental file 1

## Data Availability

All data relevant to the study are included in the article or uploaded as supplementary information.
