# Supplementary material for: Temporal trends in hospitalisations for venous thromboembolic events in England: a population-level analysis
Source: BMJ Open. 2025 Mar 29;15(3):e090301. doi: 10.1136/bmjopen-2024-090301 (PMC11956333; doi:10.1136/bmjopen-2024-090301)
Supplement: online supplemental file 1 [file bmjopen-15-3-s001.docx]

**SUPPLEMENTARY MATERIAL**

**Supplementary Figure 1.** Sensitivity analysis, using finished admission episodes rather than finished consultant episodes, to describe the hospitalisation rate per 100,000 population of hospitalisations with primary admission diagnoses of (A) VTE (DVT and PE combined), (B) PE, and (C) DVT in England between 1998 and 2022.


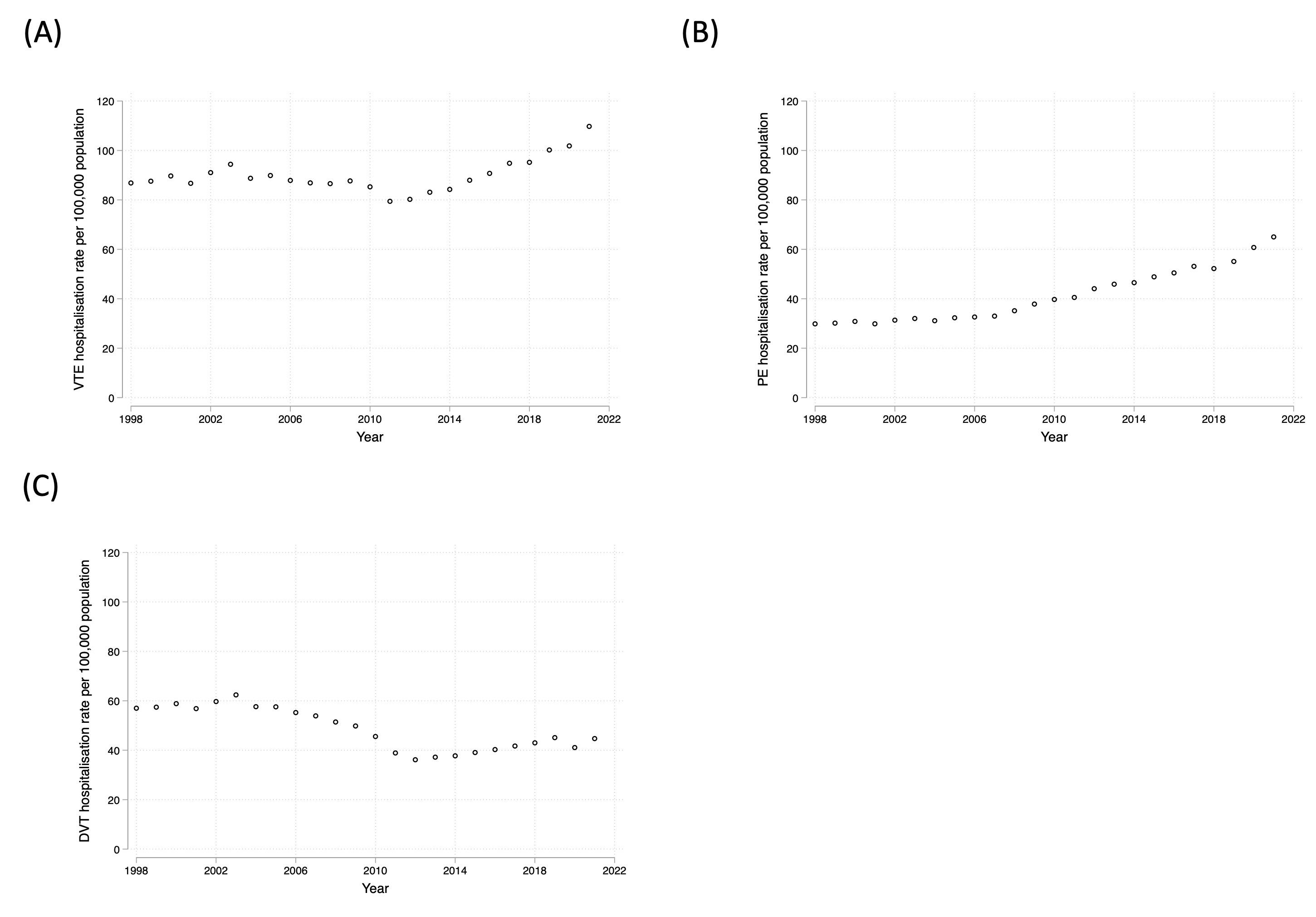


**Supplementary Figure 2**. Hospitalisation rate of (A) VTE, (B) PE and (C) DVT admissions in males vs. females between 1998 and 2022. Males are denoted by black circles, and females are denoted by grey squares.


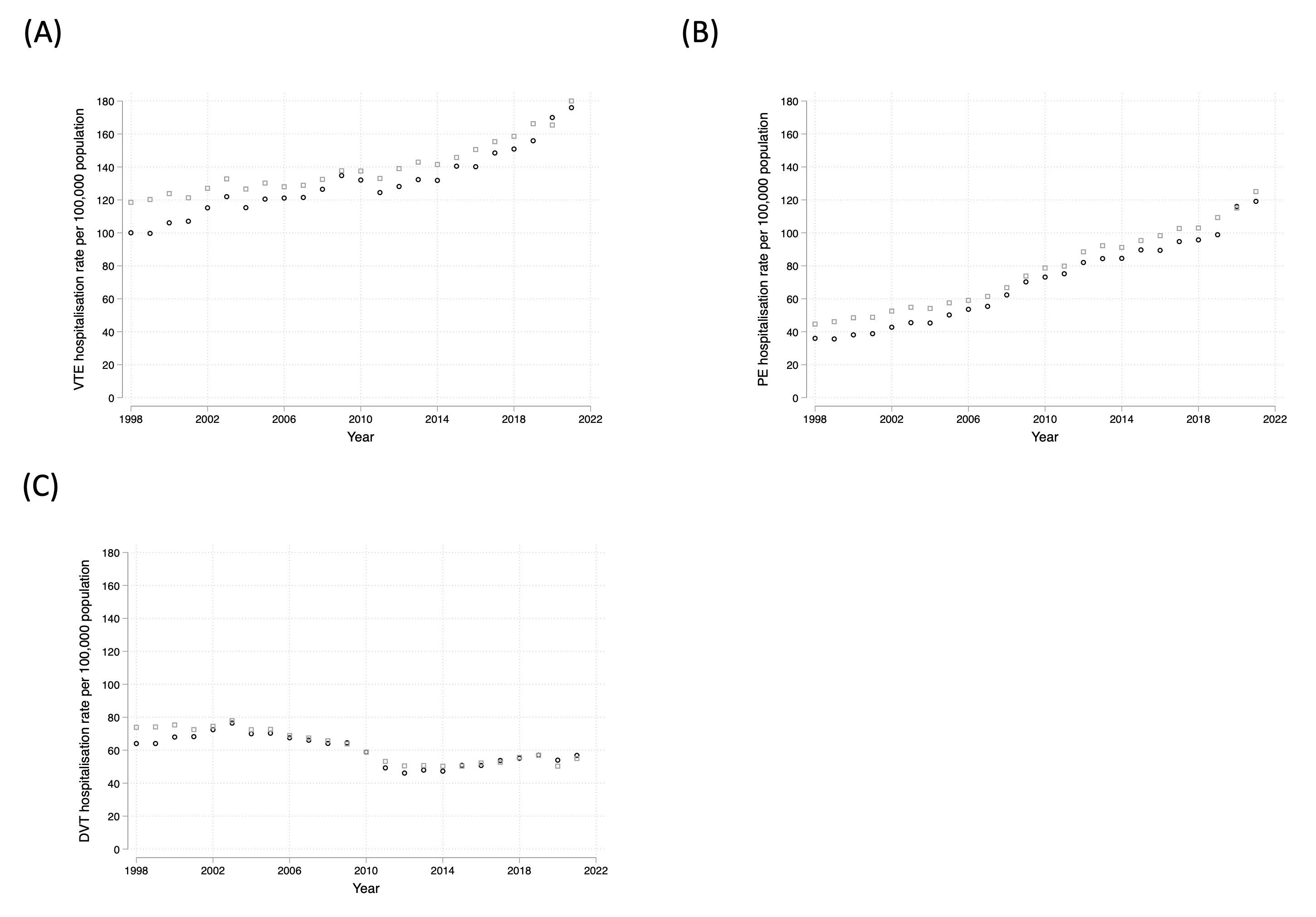


**Supplementary Figure 3.** Mean age of people hospitalised for (A) VTE, (B) PE or (C) DVT diagnoses between 1998 and 2022.


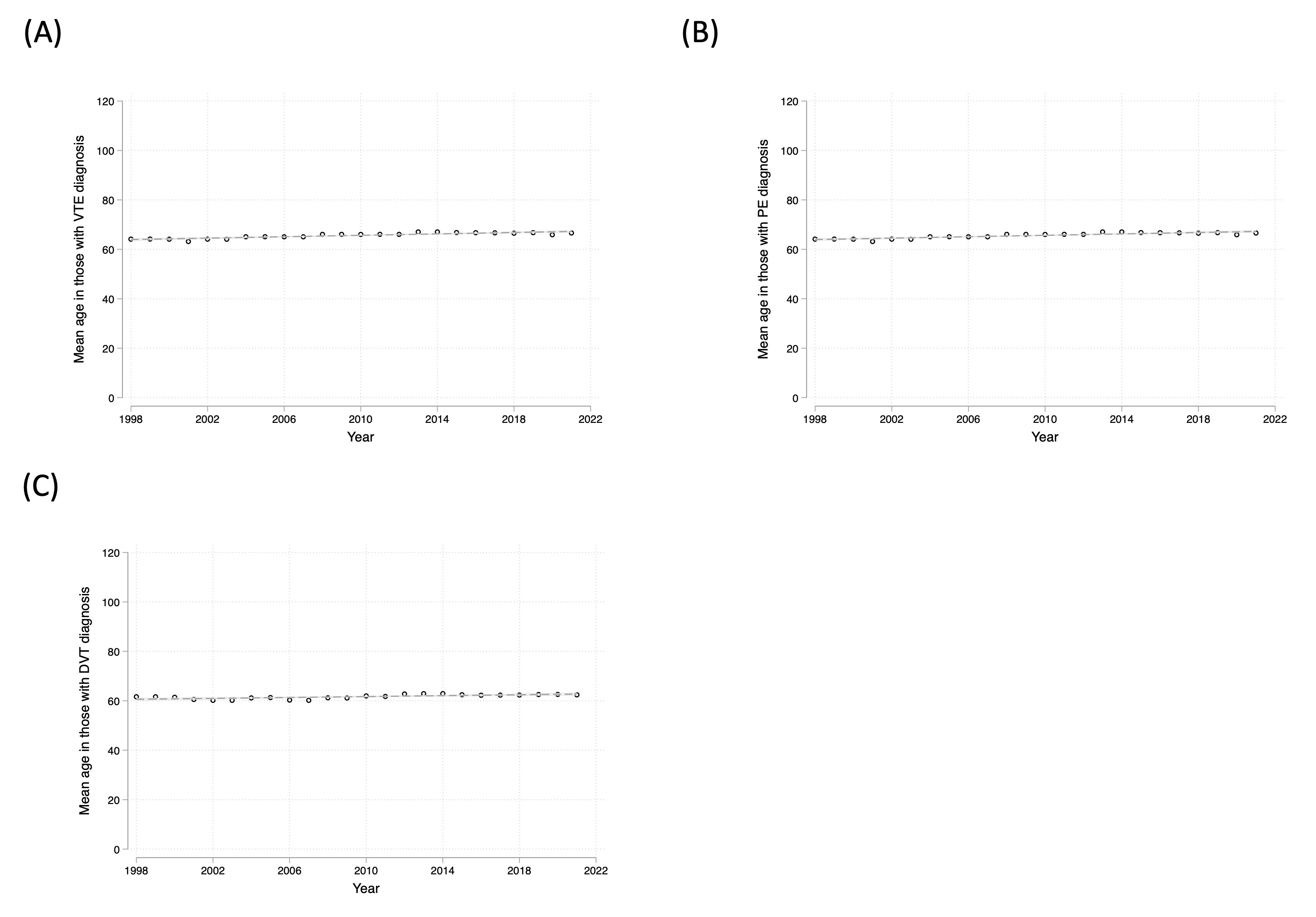


**Supplementary Figure 4.** Age-standardised rates for (A) VTE, (B) PE and (C) DVT against the year 2000 population.


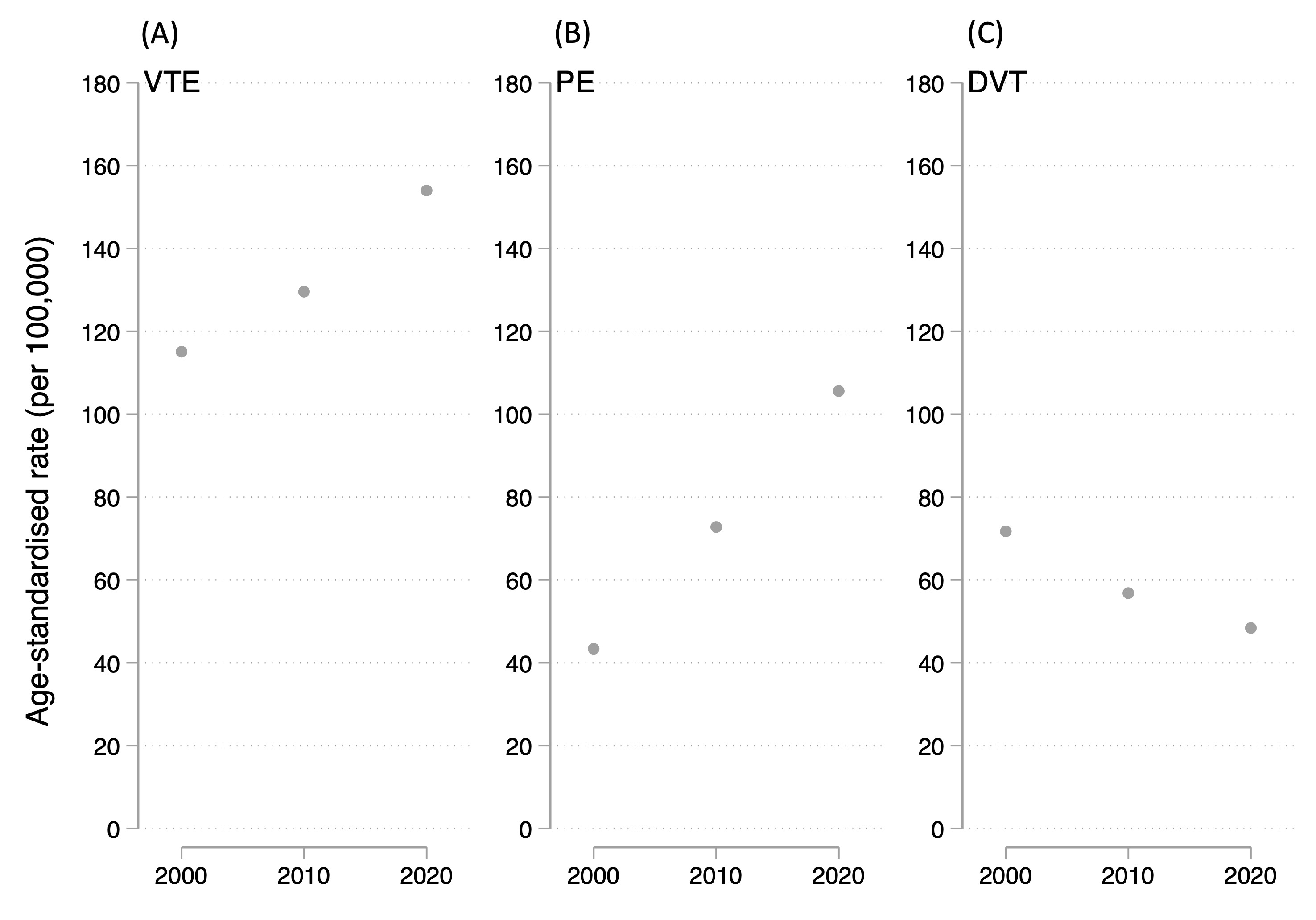


**Supplementary Table 1.** Count of finished admission episodes, and corresponding hospitalisation rate per 100,000 population, for VTE (DVT and PE combined), PE and DVT in England between 1998 and 2022.

| **Year** | **Number of VTE admission episodes** | **VTE hospitalisation rate (per 100,000 population)** | **Number of PE admission episodes** | **PE hospitalisation rate (per 100,000 population)** | **Number of DVT admission episodes** | **DVT hospitalisation rate (per 100,000 population)** |
| --- | --- | --- | --- | --- | --- | --- |
| 1998/99 | 42397 | 86.8 | 14573 | 29.9 | 27824 | 57.0 |
| 1999/00 | 42933 | 87.6 | 14781 | 30.1 | 28152 | 57.4 |
| 2000/01 | 44150 | 89.7 | 15179 | 30.8 | 28971 | 58.8 |
| 2001/02 | 42873 | 86.7 | 14766 | 29.9 | 28107 | 56.8 |
| 2002/03 | 45228 | 91.0 | 15573 | 31.3 | 29655 | 59.7 |
| 2003/04 | 47131 | 94.4 | 15983 | 32.0 | 31148 | 62.4 |
| 2004/05 | 44540 | 88.7 | 15621 | 31.1 | 28919 | 57.6 |
| 2005/06 | 45474 | 89.9 | 16347 | 32.3 | 29127 | 57.6 |
| 2006/07 | 44783 | 87.9 | 16629 | 32.6 | 28154 | 55.2 |
| 2007/08 | 44639 | 86.9 | 16948 | 33.0 | 27691 | 53.9 |
| 2008/09 | 44857 | 86.6 | 18214 | 35.2 | 26643 | 51.4 |
| 2009/10 | 45763 | 87.7 | 19763 | 37.9 | 26000 | 49.8 |
| 2010/11 | 44891 | 85.3 | 20908 | 39.7 | 23983 | 45.6 |
| 2011/12 | 42179 | 79.4 | 21525 | 40.5 | 20654 | 38.9 |
| 2012/13 | 42916 | 80.2 | 23578 | 44.1 | 19338 | 36.2 |
| 2013/14 | 44758 | 83.1 | 24725 | 45.9 | 20033 | 37.2 |
| 2014/15 | 45757 | 84.2 | 25260 | 46.5 | 20497 | 37.7 |
| 2015/16 | 48184 | 87.9 | 26777 | 48.9 | 21407 | 39.1 |
| 2016/17 | 50142 | 90.7 | 27888 | 50.5 | 22254 | 40.3 |
| 2017/18 | 52736 | 94.8 | 29541 | 53.1 | 23195 | 41.7 |
| 2018/19 | 53289 | 95.2 | 29227 | 52.2 | 24062 | 43.0 |
| 2019/20 | 56396 | 100.2 | 31009 | 55.1 | 25387 | 45.1 |
| 2020/21 | 57586 | 101.8 | 34353 | 60.7 | 23233 | 41.1 |
| 2021/22 | 62036 | 109.7 | 36757 | 65.0 | 25279 | 44.7 |

**Supplementary Table 2**. Primary admission diagnoses of VTE, PE and DVT as a percentage of all-cause hospital admissions between 1998-2022.

| ***Year*** | **All-cause admissions** | **VTE admissions** | **VTE as % of all-cause admissions** | **PE admissions** | **PE as % of all-cause admissions** | **DVT admissions** | **DVT as % of all-cause admissions** |
| --- | --- | --- | --- | --- | --- | --- | --- |
| 1998/99 | 11983893 | 53473 | 0.45 | 19739 | 0.16 | 33734 | 0.28 |
| 1999/00 | 12167574 | 54038 | 0.44 | 20093 | 0.17 | 33945 | 0.28 |
| 2000/01 | 12264677 | 56703 | 0.46 | 21379 | 0.17 | 35324 | 0.29 |
| 2001/02 | 12357360 | 56533 | 0.46 | 21705 | 0.18 | 34828 | 0.28 |
| 2002/03 | 12757656 | 60197 | 0.47 | 23699 | 0.19 | 36498 | 0.29 |
| 2003/04 | 13174480 | 63569 | 0.48 | 25062 | 0.19 | 38507 | 0.29 |
| 2004/05 | 13706765 | 60655 | 0.44 | 24951 | 0.18 | 35704 | 0.26 |
| 2005/06 | 14423506 | 63304 | 0.44 | 27205 | 0.19 | 36099 | 0.25 |
| 2006/07 | 14784581 | 63258 | 0.43 | 28611 | 0.19 | 34647 | 0.23 |
| 2007/08 | 15359062 | 64008 | 0.42 | 29877 | 0.19 | 34131 | 0.22 |
| 2008/09 | 16232579 | 66656 | 0.41 | 33231 | 0.20 | 33425 | 0.21 |
| 2009/10 | 16806196 | 70603 | 0.42 | 37333 | 0.22 | 33270 | 0.20 |
| 2010/11 | 17269882 | 70974 | 0.41 | 39987 | 0.23 | 30987 | 0.18 |
| 2011/12 | 17465425 | 68414 | 0.39 | 41176 | 0.24 | 27238 | 0.16 |
| 2012/13 | 17715046 | 71490 | 0.40 | 45626 | 0.26 | 25864 | 0.15 |
| 2013/14 | 18163101 | 74183 | 0.41 | 47594 | 0.26 | 26589 | 0.15 |
| 2014/15 | 18731987 | 74264 | 0.40 | 47734 | 0.25 | 26530 | 0.14 |
| 2015/16 | 19239608 | 78426 | 0.41 | 50696 | 0.26 | 27730 | 0.14 |
| 2016/17 | 19726907 | 80373 | 0.41 | 51894 | 0.26 | 28479 | 0.14 |
| 2017/18 | 20030870 | 84532 | 0.42 | 54919 | 0.27 | 29613 | 0.15 |
| 2018/19 | 20760699 | 86647 | 0.42 | 55626 | 0.27 | 31021 | 0.15 |
| 2019/20 | 20912276 | 90712 | 0.43 | 58636 | 0.28 | 32076 | 0.15 |
| 2020/21 | 16168689 | 94874 | 0.59 | 65389 | 0.40 | 29485 | 0.18 |
| 2021/22 | 19626344 | 100665 | 0.51 | 69064 | 0.35 | 31601 | 0.16 |

**Supplementary Table 3.** Proportion of all VTE diagnoses (primary and secondary) where VTE was listed as the primary admission diagnosis, between 2012 and 2022.

| **Year** | **Primary and secondary VTE diagnoses (hospitalisation rate per 100,000 population)** | **Primary VTE diagnoses only (hospitalisation rate per 100,000 population)** | **Proportion primary VTE diagnoses (%)** |
| --- | --- | --- | --- |
| 2012/13 | 251.9 | 133.6 | 53.0 |
| 2013/14 | 264.9 | 137.7 | 52.0 |
| 2014/15 | 269.8 | 136.7 | 50.7 |
| 2015/16 | 290.8 | 143.1 | 49.2 |
| 2016/17 | 305.7 | 145.4 | 47.6 |
| 2017/18 | 325.4 | 152.0 | 46.7 |
| 2018/19 | 338.4 | 154.8 | 45.7 |
| 2019/20 | 349.1 | 161.2 | 46.2 |
| 2020/21 | 393.3 | 167.8 | 42.7 |
| 2021/22 | 401.2 | 178.1 | 44.4 |

**Supplementary Table 4.** Proportion of all PE diagnoses (primary and secondary) where PE was listed as the primary admission diagnosis, between 2012 and 2022.

| **Year** | **Primary and secondary PE diagnoses (hospitalisation rate per 100,000 population)** | **Primary PE diagnoses only (hospitalisation rate per 100,000 population)** | **Proportion primary PE diagnoses (%)** |
| --- | --- | --- | --- |
| 2012/13 | 147.8 | 85.3 | 57.7 |
| 2013/14 | 156.0 | 88.4 | 56.6 |
| 2014/15 | 159.8 | 87.9 | 55.0 |
| 2015/16 | 173.6 | 92.5 | 53.3 |
| 2016/17 | 183.7 | 93.9 | 51.1 |
| 2017/18 | 195.2 | 98.7 | 50.6 |
| 2018/19 | 201.3 | 99.4 | 49.4 |
| 2019/20 | 211.4 | 104.2 | 49.3 |
| 2020/21 | 265.1 | 115.6 | 43.6 |
| 2021/22 | 267.0 | 122.2 | 45.7 |

**Supplementary Table 5.** Proportion of all DVT diagnoses (primary and secondary) where DVT was listed as the primary admission diagnosis, between 2012 and 2022.

| **Year** | **Primary and secondary DVT diagnoses (hospitalisation rate per 100,000 population)** | **Primary DVT diagnoses only (hospitalisation rate per 100,000 population)** | **Proportion primary DVT diagnoses (%)** |
| --- | --- | --- | --- |
| 2012 /13 | 104.1 | 48.3 | 46.4 |
| 2013/14 | 108.9 | 49.4 | 45.3 |
| 2014/15 | 110.0 | 48.8 | 44.4 |
| 2015/16 | 117.2 | 50.6 | 43.2 |
| 2016/17 | 122.0 | 51.5 | 42.2 |
| 2017/18 | 130.3 | 53.2 | 40.9 |
| 2018/19 | 137.1 | 55.4 | 40.4 |
| 2019/20 | 137.7 | 57.0 | 41.4 |
| 2020/21 | 128.2 | 52.1 | 40.7 |
| 2021/22 | 134.1 | 55.9 | 41.7 |
