# Supplementary material for: Temporal trends in hospitalisations for venous thromboembolic events in England: a population-level analysis
Source: BMJ Open. 2025 Mar 29;15(3):e090301. doi: 10.1136/bmjopen-2024-090301 (PMC11956333; doi:10.1136/bmjopen-2024-090301)
Supplement: Supplementary file 2 [file bmjopen-2024-090301.reviewer_comments.pdf]

PEER REVIEW HISTORY

BMJ Open publishes all reviews undertaken for accepted manuscripts. Reviewers are asked to complete a checklist review form and are provided with free text boxes to elaborate on their assessment. These free text comments are reproduced below.

ARTICLE DETAILS

Title (Provisional)

Temporal trends in hospitalisations for venous thromboembolic events in England: a population-level analysis

Authors

Hughes, Mark; Russell, Mark D; Roy, Ritika; Mehta, Daksh; Norton, Sam; Atzeni, Fabiola; Galloway, James B

VERSION 1 - REVIEW

**Reviewer**	**1**
**Name**	**Mumoli, Nicola**
**Affiliation**	**Department of Internal Medicine, Magenta Hospital, Italy**
**Date**	**17-Jul-2024**
**COI**	**none**

Thanks very much for this very interesting and informative article. I feel that overall the paper was well written and without significant flaws. The study objectives and the methods section are clearly defined, the article is easily readable, and the topic is relevant to the readership. Limitations are adequately addressed. Conclusions are appropriate for the scope of the study. The paper is formally correct and it is clear its clinical relevance, and what this article should add to the body of knowledge on this topic.

**Reviewer**	**2**
**Name**	**Bar-Or, David**
**Affiliation**	**Swedish Medical Center, Trauma Research Department**
**Date**	**23-Oct-2024**
**COI**	**I do not have competing interests**

Hughes et al in manuscript bmjopen-2024-090301 report on an epidemiological observational study of the incidence of VTE in the UK between 1998 and 2022 using aggregate hospitalization data from the NHS Digital Hospital Episode Statistics dataset.

They conclude that hospitalizations for VTE increased during that time period significantly and driven by large increase in hospitalizations for Pes (increase of 202%) while decreasing for DVT (decrease of 19.1%). They attribute the changes possibly to better prehospital protocols and education for DVT and perhaps the greater use of biomarkers and CTA for diagnosing PE. Obesity, although a risk factor for VTE is somewhat questionable as it cannot explain the disparity between PE and DVT incidence changes.

Further exploring the etiology of VTE in this population, for example Trauma patients, premorbid conditions such as cardiovascular diseases and risk factors, use of anticoagulants or antiplatelets in subgroup analysis could be informative.

A potential analysis using joint point statistics could be helpful in understanding temporal causes of the changes observed.

**Reviewer**	**3**
**Name**	**Conrad, Nathalie**
**Affiliation**	**KU Leuven, Medical Sciences Division**
**Date**	**06-Nov-2024**
**COI**	**N/A**

Thank you for this interesting study.

As I understand, authors did not have access to patient-level data. As such the data presented are not incidence rates. Incidence rates would be the number of individuals newly diagnosed with a condition. In this study one individual can be admitted with VTE on several occasions and authors would not have the possibility of distinguishing between first and repeat admissions. In that case, the data presented are "hospitalisation rates" and not "incidence rates". This means the wording should be changed (to "hospitalisation rates"). Also the discussion / interpretation of findings should make sure this is clear (eg. When comparing to studies that have investigated the incidence of VTE) and discuss how recurrent events may have influenced the observed increases / decreases in the number of hospitalisations.

Similarly, "age at presentation" would be better reworded as "age at hospitalisation".

In the methods, would authors please clarify the difference between "finished consultant episodes" and "admission episodes". Can a patient be diagnosed with VTE several times (ie. in several consultant episodes) as part of one same admission? And if so, how does this impact the present findings?

I could not find out how “age-standardised rates” were calculated. This should be clarified in the methods.

In the results, age-stratified rates rely on very broad age-groups. It would be helpful to see data by 5 or 10-year age bands.

In the discussion, authors claim that “population ageing accounted for only a small proportion of the changes observed in the rates of DVT, PE and VTE admissions in our study”. That may well be true, but authors would need to clarify how they arrive to that conclusion in the results.

It would be interesting to discuss how these findings compare to the incidence / number of hospitalisations of other cardiovascular diseases, such as MI which has declined considerably over the same period.

Similarly, it would be interesting to discuss how changes in prescriptions of anticoagulants after a first VTE or AF over the study period may have influenced the present findings.

VERSION 1 - AUTHOR RESPONSE

Reviewer: 1

Reviewer comment: Thanks very much for this very interesting and informative article. I feel that overall the paper was well written and without significant flaws. The study objectives and the methods section are clearly defined, the article is easily readable, and the topic is relevant to the readership. Limitations are adequately addressed. Conclusions are appropriate for the scope of the study. The paper is formally correct and it is clear its clinical relevance, and what this article should add to the body of knowledge on this topic.

Authors’ response: Thank you very much for their kind comments.

Reviewer: 2

Reviewer comment: Hughes et al in manuscript bmjopen-2024-090301 report on an epidemiological observational study of the incidence of VTE in the UK between 1998 and 2022 using aggregate hospitalization data from the NHS Digital Hospital Episode Statistics dataset. They conclude that hospitalizations for VTE increased during that time period significantly and driven by large increase in hospitalizations for Pes (increase of 202%) while decreasing for DVT (decrease of 19.1%). They attribute the changes possibly to better prehospital protocols and education for DVT and perhaps the greater use of biomarkers and CTA for diagnosing PE.

Authors’ response: Thank you very much for their kind comments.

Reviewer comment: A potential analysis using joint point statistics could be helpful in understanding temporal causes of the changes observed.

Authors’ response: We appreciate the reviewer’s suggestion to consider joinpoint analysis. However, given the limited number of data points and the annualised aggregation of data, joinpoint regression was not entirely feasible in this context. Instead, we have implemented

joinpoint regression using a segmented regression approach, with an automated search across breakpoints to explore temporal changes while accounting for the data's limitations. This has identified that non-linear trends fit better than a linear trend for most models; however, there is not clear 'cause' associated with the breakpoints identified.

Reviewer comment: Obesity, although a risk factor for VTE is somewhat questionable as it cannot explain the disparity between PE and DVT incidence changes. Further exploring the etiology of VTE in this population, for example Trauma patients, premorbid conditions such as cardiovascular diseases and risk factors, use of anticoagulants or antiplatelets in subgroup analysis could be informative.

Authors' response: We agree entirely with the reviewer on this point. We have now adjusted the relevant discussion paragraph (page 9, line 232) to make clear that changing prevalence of obesity would not explain the observed disparity between PE and DVT hospitalisations. We also agree with the author that it would be very informative to perform subgroup analyses by the risk factors suggested; however, unfortunately due to the aggregated nature of the HES dataset, it is not possible to separate out individuals with VTE according to their underlying risk factors. This is an area of interest that we would like to explore in future studies with individual-level patient data.

Reviewer: 3

Thank you for this interesting study.

Reviewer comment: As I understand, authors did not have access to patient-level data. As such the data presented are not incidence rates. Incidence rates would be the number of individuals newly diagnosed with a condition. In this study one individual can be admitted with VTE on several occasions and authors would not have the possibility of distinguishing between first and repeat admissions. In that case, the data presented are "hospitalisation rates" and not "incidence rates". This means the wording should be changed (to "hospitalisation rates"). Also the discussion / interpretation of findings should make sure this is clear (eg. When comparing to studies that have investigated the incidence of VTE) and discuss how recurrent events may have influenced the observed increases / decreases in the number of hospitalisations. Similarly, "age at presentation" would be better reworded as "age at hospitalisation".

Authors' response: Thank you very much to the reviewer for these comments. We agree entirely that hospitalisation rate would be more appropriate than incidence rate, for the reasons mentioned by the reviewer. We have amended the manuscript accordingly. We have now made clear in the discussion limitations section that, due to the aggregated nature of the dataset, we are unable to separate out incident from recurrent events.

Reviewer comment: In the methods, would authors please clarify the difference between "finished consultant episodes" and "admission episodes". Can a patient be diagnosed with VTE several times (ie. in several consultant episodes) as part of one same admission? And if so, how does this impact the present findings?

Authors' response: That is correct: while most admissions will be represented by a single finished consultant episode, in more complex admissions where patients are transferred between different consultants, there can be multiple finished consultant episodes (FCE) during a single admission. This could lead to multiple finished consultant episodes with primary diagnostic codes for VTE, despite it representing a single admission. While much of the aggregated HES dataset is structured around FCE events, this was our rationale for performing sensitivity analyses using admission episodes rather than FCE, which yielded very similar trends. We have now clarified this in our methods section.

Reviewer comment: I could not find out how "age-standardised rates" were calculated. This should be clarified in the methods. In the results, age-stratified rates rely on very broad age-groups. It would be helpful to see data by 5 or 10-year age bands.

Authors' response: Thank you for these comments. We have now included further information on how we calculated age-stratified and age-standardised rates were calculated in our methods section. We agree that with the reviewer that, ideally we would have presented age-stratified rates using narrower age bands. Unfortunately, however, earlier HES datasets only presented admission events separated into these broader age bands, which precluded these analyses. We have now added this as a limitation in our discussion.

Reviewer comment: In the discussion, authors claim that "population ageing accounted for only a small proportion of the changes observed in the rates of DVT, PE and VTE admissions in our study". That may well be true, but authors would need to clarify how they arrive to that conclusion in the results.

Authors' response: Thank you, we have now amended this paragraph, highlighting that our age-standardised rates yielded similar trends to our primary (non-standardised) analyses, in addition to presenting data on trends in mean age at hospitalisation, and age-stratified rates, in our results section.

Reviewer comment: It would be interesting to discuss how these findings compare to the incidence / number of hospitalisations of other cardiovascular diseases, such as MI which has declined considerably over the same period.

Authors' response: Thank you for this suggestion. We have now referred to this in our discussion section.

Reviewer comment: Similarly, it would be interesting to discuss how changes in prescriptions of anticoagulants after a first VTE or AF over the study period may have influenced the present findings.

Authors' response: This is a very interesting point. While we were unable to analyse differences in anticoagulant use in this particular study, we have now mentioned this in our discussion section and highlight it as a point for further study.

VERSION 2 - REVIEW

**Reviewer**	**3**
**Name**	**Conrad, Nathalie**
**Affiliation**	**KU Leuven, Medical Sciences Division**
**Date**	**16-Jan-2025**
**COI**	

Thank you for revising your manuscript following reviewer comments. A few points require further clarification.

1- I could not find out how authors calculated “mean age at VTE hospitalisation”, if patient-level data was not available to them. Could authors please explain this in their methods.

2- Truncated y-axis plots can be misleading, could authors either start plots at 0 clearly label the axis as being truncated.

3- Authors state that hypentension prevalence in the UK has increased over their study period. This is not true, hypertension has remained broadly stable / declined modestly. The reference included by the authors does not actually describe hypertension trends. See the Health Survey England - https://digital.nhs.uk/data-and-information/publications/statistical/health-survey-for-england/2021-part-2/adult-health-hypertension

4- Authors state “it is unclear whether changes in prescribing practices after first VTE events or in the context of atrial fibrillation may have influenced hospitalisations.” I find this to be a rather simplistic statement. Data on prescription rates after AF and VTE is well described. Overall the proportion of individuals with VTE on anticoagulants (considering either DOAC and Warfarin) seems to have declined slightly over the study period, see for eg. https://www.bmj.com/content/385/bmj-2023-078523 Figure S7

5- I am still missing a discussion of how trends reported in this study are influenced by both first events (ie. incidence) and recurrent hospitalisations. Ie. are we seeing more patients being diagnosed with VTE (which would point towards missed opportunities in risk factor management / primary prevention)? or are we seeing the same number of patients with VTE, only that these patients are now hospitalised more often (which would rather point towards missed opportunities in treatement/secondary prevention)? This is a key question here, and if authors cannot answer this question with the data that they have, then this should at least be mentioned in the context of existing literature.

VERSION 2 - AUTHOR RESPONSE

Thank you very much to the reviewer for their comments, which we have addressed below.

Reviewer comment: I could not find out how authors calculated “mean age at VTE hospitalisation”, if patient-level data was not available to them. Could authors please explain this in their methods.

Authors’ response: Thank you for these comments. The mean age at hospitalisation is provided in the HES datasets for each year. This clarification has now been added to the manuscript under the methods sections: “The mean age was provided within the HES datasets for each year”.

Reviewer comment: Truncated y-axis plots can be misleading, could authors either start plots at 0 clearly label the axis as being truncated.

Authors’ response: We agree with the reviewer that starting the plots at 0 would be less misleading, and we have amended the truncated figures accordingly.

Reviewer comment: Authors state that hypertension prevalence in the UK has increased over their study period. This is not true, hypertension has remained broadly stable / declined modestly. The reference included by the authors does not actually describe hypertension trends. See the Health Survey England - https://digital.nhs.uk/data-and-information/publications/statistical/health-survey-for-england/2021-part-2/adult-health-hypertension

Authors’ response: Thank you for providing this reference. We have now amended this in our manuscript: “Hypertension prevalence remained broadly stable between 2003 and 2014 and decreased modestly until 2019, suggesting this was unlikely to have influenced our observed trends [18]”. We used the reference kindly recommended by the reviewer.

Reviewer comment: Authors state “it is unclear whether changes in prescribing practices after first VTE events or in the context of atrial fibrillation may have influenced hospitalisations.” I find this to be a rather simplistic statement. Data on prescription rates after AF and VTE is well described. Overall the proportion of individuals with VTE on anticoagulants (considering either DOAC and Warfarin) seems to have declined slightly over the study period, see for eg. https://www.bmj.com/content/385/bmj-2023-078523 Figure S7

Authors’ response: Thank you for providing this information and reference, which we read with interest. We have now provided this information for readers in our discussion, along with the reference and an interpretation of how this might have contributed to the observed findings: “Additionally, DOAC use has increased significantly, from 0% in 2000-2002 to 47.3% in 2017-2019, whereas warfarin experienced a sharp decrease in use over the same time

period (from 58.1% to 6.6%, respectively), resulting in a slight decline in the overall proportion of individuals with VTE who were initiated on anticoagulants [22]. A reduction in the proportion of individuals with VTE who received anticoagulant therapy could potentially have contributed to the temporal trends observed in our study, for example through recurrent VTE events and/or progression of DVT to PE. While we were unable to explore trends in the proportion of hospitalisation events in our study that were from incident versus recurrent VTE events due to the aggregated data sources utilised, this certainly warrants further investigation in future studies."

Reviewer comment: I am still missing a discussion of how trends reported in this study are influenced by both first events (ie. incidence) and recurrent hospitalisations. Ie. are we seeing more patients being diagnosed with VTE (which would point towards missed opportunities in risk factor management / primary prevention)? or are we seeing the same number of patients with VTE, only that these patients are now hospitalised more often (which would rather point towards missed opportunities in treatment/secondary prevention)? This is a key question here, and if authors cannot answer this question with the data that they have, then this should at least be mentioned in the context of existing literature.

Authors' response: The relative contribution of first vs. recurrent VTE events is important to understanding the trends highlighted in our study. Unfortunately, we are unable to answer this in the data available to us. We have now included this as a key limitation, and we now mention the important points raised by the reviewer about the need for a better understanding of the relative contribution of missed opportunities for primary and/or secondary prevention: "Similarly, we were unable to separate out incident VTE admission events from recurrent VTE admissions, which precluded further investigation of whether there were missed opportunities for primary VTE prevention (e.g. due to risk factor management) and/or missed opportunities for secondary prevention (e.g. due to inadequate treatment)."

VERSION 3 - REVIEW

**Reviewer**	**3**
**Name**	**Conrad, Nathalie**
**Affiliation**	**KU Leuven, Medical Sciences Division**
**Date**	**24-Feb-2025**
**COI**	

Thank you for addressing all questions thoroughly.

VERSION 3 - AUTHOR RESPONSE

Reviewer: 3

Reviewer comment: Thank you for addressing all questions thoroughly.

Authors' response: Thank you very much for their comments.
